# Senior Resident Training on Educational Principles (STEP): A Proposed Innovative Step from a Developing Nation

**DOI:** 10.3352/jeehp.2010.7.3

**Published:** 2010-12-01

**Authors:** Satendra Singh

**Affiliations:** Department of Physiology and Medical Education Unit, University College of Medical Sciences, University of Delhi, Delhi, India.

**Keywords:** Resident-as-Teacher, Medical Education Unit, Pedagogical Intervention, Adult Training, Transfer of Training, Andragogy, Senior Residents, Medical Residency

## Abstract

Resident-as-teacher courses are pretty common in Western medical schools however they are a rarity in Asian and developing countries. The current report is a scholarly analysis of a three day orientation program for senior residents in order to improve their functioning by providing new template either for supplementing basic workshops for faculty or to advocate a change in system. The experience gained by Medical Education Unit of University College of Medical Sciences can be used to conduct training breeding grounds at national or regional levels. Resident as teachers educational interventions need to be designed taking into account their impact on education system.

## INTRODUCTION

Senior residents are entrusted with the responsibility of 80% of teaching of interns, medical students as well as patients regardless of their future career plans [1]. Nearly two-thirds of residents receive more than 40% of their education from other fellow residents [2]. Studies also show that residents conduct more teaching at the bedside compared to attending [3]. Despite their significant teaching responsibilities, they receive no formal instruction on how to teach effectively. Resident-as-teacher courses are flourishing in Western medical schools however there is a paucity of published information from Asian and developing countries [4].

The Medical Council of India (MCI) has made it mandatory for Medical Education Units (MEU) in India to conduct basic workshops on medical education technologies for younger faculty [5]. The actual implementation is however sporadic. The objective of the current report is to describe the MEU, University College of Medical Sciences (UCMS), Delhi's initiative to propose a resident-as-educator program as a first step in a strategy to develop educational intervention for residents' teaching skills. The secondary objective was to complement MEU's mandate of training younger faculty by sensitizing residents who are going to be the future faculty members.

## METHODS

### Study setting

The MEU is the educational entity that conducts programs for undergraduate, postgraduate, residents and faculty members at UCMS [6]. A three day orientation course (workshop) for senior residents was designed by MEU, UCMS and was named Senior Resident Training on Educational Principles (STEP). The objectives of STEP was to provide the residents a repertoire of pedagogical practices from which to plan, implement and evaluate their own teaching to engage students in the learning process, to demonstrate competence and skill in understanding and using a range of assessment methods, to give feedback in an appropriate manner and to employ goal-seeking behavior and team-work in medical education, and as life skills.

### Study design

The assessment needs of participants were performed by verbal feedback from focus groups and performance review. Program director (coordinator of medical education unit) and program coordinator (author) led the dual moderator focus group. Verbal feedbacks were recorded by later and transformed into written statements by frequent group meetings while preparation of the blueprint. A questionnaire survey is a better tool for performing needs assessment and we consider its lack as our limitation. The principles of adult learning were followed and voluntary residents were asked to register online. To facilitate better group interaction only twenty participants on first-cum-first basis were registered for STEP. The participants were residents from physiology, biochemistry, pediatrics, surgery, pharmacology, pathology, dermatology, forensic medicine, anesthesiology and ophthalmology. The course content and methodology adopted for STEP is given in Table 1. The three day course content was largely modified as per rankings given to themes to be used in a resident-as-teacher educational intervention having largest sample size reported till date [4].

Transfer of training model was deliberately employed to give chance to the earlier trained faculty at our institute to empower their skills in conjunction with experienced MEU core members to create a receptive environment for learning and evolving [7, 8].

Pre and post tests are at best a measure of short term memory. More often than not they assess factual knowledge change and as such were not used in STEP. Rather than focusing on output, stress was given on outcome and the only evaluation instrument collected at the end of the workshop was written program feedback.

## RESULTS

**Fig. 1:**
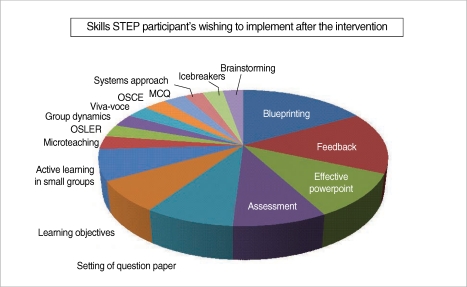
Pedagogical skills residents want to implement after the intervention. STEP, Senior Resident Training on Educational Principles; MCQ, multiple-choice question; OSCE, objective structured clinical exams; OSLER, objective structured long examination record.

Senior resident's self-assessment in the form of program feedback is depicted in Table 2 and the pedagogical skills residents wish to implement after this intervention is shown in Fig. 1. Residents reported positive changes and self-confidence in attitude towards teaching. They predominantly wish to apply the skills of blueprinting, feedback, effective powerpoint and use of evaluation tools more than the other strategies.

## DISCUSSION

Residents are frequently identified by medical students as their most frequent and memorable teachers [9]; They spend more time in direct contact with the health care institutions in-training personnel than many specialists or attending physicians, "and they influence greatly the hidden curriculum of the educational activities in daily clinical care" [4]. Despite influencing the majority of the unstructured 'informal curriculum' many of them teaches ineffectively [10-12]. There is no educational program tailored to their need to teach them pedagogical practices. Therefore it is indeed pertinent to optimize the teaching skills of residents and provide them with formal teacher training.

The philosophy behind our faculty development is exactly the same as that of pedagogical active learning strategy that students "learn best by doing, not by watching or listening" [13]. The same holds true for andragogy. We empowered our trained faculty members from previous workshop to act as facilitators to sensitize residents. Repeating this educational intervention will help MEU UCMS in identifying new facilitators from the resident group, a factor which will contribute to resolving the acute shortage of medical educationists. A study from Pakistan found residents to be an effective supplement to faculty members for facilitation of problem-based learning sessions [14].

In India, our residents are a breed of passionate fliers without adequate wings. The current report is the result of a first program for residents at our institute. The pedagogic effectiveness of STEP was attributed to a resident-centered, learning format with small group sessions. The participants liked non-competitive interactions among junior faculty members and appreciated participation of MEU faculty in a non-dominant role. Our findings are similar to other authors with a preference for interactive small group sessions with facilitators [4, 14-19]. Further researches are advocated so that medical schools are inclined to move from opinion-based tradition to evidence-based education.

### Limitations

We did not used pre- and post-intervention outcome comparison method which may be considered by some as a limitation, as it only evaluates short-term memory. Sampling included residents from our own institute but as we are not aware of any other published work from India so it should be considered as an encouraging beginning with much scope for flexibility. Although resident self-confidence is important, self-assessment may not represent actual skills obtained and we consider it as a limitation.

### Future directions

The literature suggests that lasting effects of interventions to improve teaching are associated with opportunities for follow-up in the teacher's own setting [20]. To address this need, the approaches proposed are to assign each resident a teaching mentor [9] and to assign them as facilitators in next resident workshop.

### Conclusions

Senior residents in India are not given adequate training to choose appropriate methodology for their future career progression. However catching them young can give them a sound foundation for honing their skills as a future faculty. Pedagogical competence is unlikely to be achieved by a single course and the need for reiteration in the form of repeated practice, feedback and self-reflection is imperative. Research also is needed to assess the lasting effect of resident teaching courses and the impact of brief follow-up sessions to reinforce skills.

## Figures and Tables

**Table 1:**
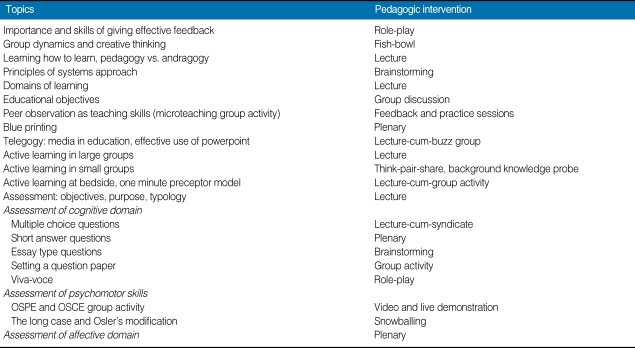
Program design for Senior Resident Training on Educational Principles (STEP)

OSPE, objective structured practical examination; OSCE, objective structured clinical exams.

**Table 2:**
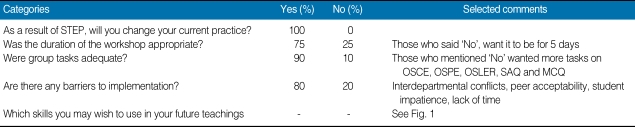
Feedback responses (n = 20)

STEP, Senior Resident Training on Educational Principles; OSCE, objective structured clinical exams; OSPE, objective structured practical examination; OSLER, objective structured long examination record; SAQ, short answer question; MCQ, multiple-choice question.
